# Dental microwear texture analysis as a tool for dietary discrimination in elasmobranchs

**DOI:** 10.1038/s41598-021-81258-9

**Published:** 2021-01-28

**Authors:** Laura J. McLennan, Mark A. Purnell

**Affiliations:** 1 Centre for Palaeobiology Research, School of Geography, Geology and the Environment, University of Leicester, Leicester, LE1 7RH UK; 2 Present Address: Department of Environmental Science, University of Derby, Derby, DE22 1GB UK

**Keywords:** Conservation biology, Ecosystem ecology, Ichthyology, Marine biology, Palaeontology

## Abstract

As abundant and widespread predators, elasmobranchs play influential roles in food-web dynamics of marine communities. Clearly, these trophic interactions have significant implications for fisheries management and marine conservation, yet elasmobranch diet is relatively understudied; for the majority of species little or no quantitative dietary data exist. This reflects the difficulties of direct observation of feeding and stomach contents analysis in wild elasmobranchs. Here, by quantifying the 3D surface textures that develop on tooth surfaces as a consequence of feeding, we show that tooth microwear varies with diet in elasmobranchs, providing a new tool for dietary analysis. The technique can be applied to small samples and individuals with no gut contents, and thus offers a way to reduce the impact on wild elasmobranch populations of analysing their dietary ecology, especially relevant in conservation of endangered species. Furthermore, because microwear accumulates over longer periods of time, analysis of texture overcomes the ‘snapshot bias’ of stomach contents analysis. Microwear texture analysis has the potential to be a powerful tool, complementing existing techniques such as stable isotope analysis, for dietary analysis in living and extinct elasmobranchs.

## Introduction

As abundant and widespread predators, elasmobranchs play influential roles in food-web dynamics of marine communities^1^. These trophic interactions have significant implications for fisheries management and marine conservation, yet elasmobranch diet is relatively understudied. For the majority of species little or no quantitative dietary data exist (e.g. Refs.^1–3^). Systematic direct observation of wild feeding in large marine predators is difficult, but the difficulty and expense of stomach contents analysis is also a factor in this deficiency of dietary data. Problems relating to stomach contents analysis are a consequence of the large numbers of individuals required for robust analysis (e.g. Ref.^4^) and the difficulties in obtaining this data for many elasmobranchs^3^. Furthermore, stomach contents provide only a ‘snapshot’ view of diet over the few hours prior to capture^5^, and are subject to other inherent biases including: elevated counts of prey with “hard parts” in relation to those without; elevated capture rates of actively foraging (‘hungry’) individuals; and inversion of stomachs due to distress resulting in loss of contents before they can be analysed^3,6–8^. Because large sample sizes are required, stomach contents analysis based on lethal sampling can also be problematic for species with threatened conservation status^9^.

More recently, additional methods have been employed to examine diet in elasmobranchs. Stable isotope analysis, particularly of δ^13^C and δ^15^N, can be used to estimate an organism’s foraging location and trophic position relative to that of others in the same food web. The approach avoids many of the pitfalls of stomach contents analysis as it provides time integrated dietary information based on assimilated biomass^7^ and can also be non-lethal and minimally invasive. It is not, however, without methodological limitations^10,11^. Stable isotope analysis provides only a measure of the relative trophic position of a species within a specific trophic web, rather than actual diet, making comparison of individuals or populations from geographically distant areas difficult unless the isotopic composition of food items in their respective trophic webs has been characterised. In addition, multiple dietary combinations can result in the same δ^13^C and δ^15^N values^7,12,13^. Quantitative analysis of fatty acid signatures^14^, and genetic tools to identify specific prey species have also been developed^15,16^.

Dental microwear texture analysis (DMTA) represents an additional, potentially powerful tool for dietary discrimination and investigation of dietary ecology in elasmobranchs. Although the method is widely applied to terrestrial mammals^17–20^, and is starting to be applied to reptiles^21,22^, teleost fishes^23,24^, and cetaceans^25^ it has not previously been applied to elasmobranchs. We present here the first evidence that three-dimensional textures of tooth microwear vary with diet in sharks.

DMTA is based on quantitative analysis of the sub-micrometre scale three-dimensional textures of wear that form on tooth surfaces during food consumption. The approach offers several advantages: it provides direct evidence of tooth use that is independent of functional analyses based on morphology of the jaws and teeth; dietary signals accumulate over longer timescales than stomach contents, avoiding the ‘snapshot’ problem^5,24^; it can detect subtle dietary differences between individuals and populations, even when sample sizes are small^24,26,27^. The method is also highly applicable to fossils^19,28,29^ and to specimens that are not amenable to stomach contents analysis, and in contrast to stable isotope analysis it provides evidence of the nature of food rather than the relative trophic level at which an organism is feeding.

Our analysis is based on the Sand Tiger shark, *Carcharias taurus*. This species is an ideal model to investigate the use of tooth microwear texture analysis for dietary discrimination in elasmobranchs. Because it survives well in captivity it is a relatively common species in aquaria (and thus teeth from individuals with controlled diets are available). Once relatively common in the wild, *C. taurus* is now one of the most threatened elasmobranchs in the world^30^, and efforts to understand its ecology, linked to conservation priorities, have resulted in detailed dietary analyses of wild populations^30–32^. Wild *C. taurus* consume mainly teleosts and elasmobranchs, with the proportions of each varying with geographical differences in prey distribution and with *C. taurus* ontogeny. The available evidence^30–32^ indicates that diet differs with size: the range of dietary items is greater in larger individuals, and in general, compared to smaller *C. taurus*, individuals exceeding approximately 2 m in total length consume more elasmobranchs (particularly benthic species). These differences provide us with the framework within which to evaluate whether microwear texture analysis can discriminate between individuals with different diets.

The focus of this study is to test the hypothesis that tooth microwear textures vary with diet in *C. taurus*. Unlike terrestrial mammals, the group upon which most studies of microwear texture are based, the polyphyodont dentition means that it is not possible to sample, in multiple individuals, the same location on a homologous wear facet, on the homologous cusp of a homologous tooth. Consequently, we also tested the subsidiary hypotheses that non-dietary variation in microwear texture between samples from different parts of a tooth is greater than variation between individuals with different diets.

Because our approach is non-destructive, acquires data from teeth, and requires only a small number of samples, it has a wide range of potential applications, including conservation of endangered elasmobranch species, and palaeontological investigation of extinct elasmobranchs. In combination with other approaches that capture dietary information on different timescales^33^, it provides independent data that could be used as part of a multiproxy approach to dietary analysis in elasmobranchs.

## Results

We tested the hypothesis that tooth microwear textures vary with diet in *C. taurus* through ANOVA. The results demonstrate that non-dietary variation in microwear texture between samples from different parts of a tooth is less than variation between individuals with different diets (Tables 1, 2, 3). Comparison of within tooth samples (2a) with data sampled from multiple teeth in specimens 1–4 reveals that 2a does not differ in any parameters from specimen 2; it differs in two parameters from specimen 3, and differs from specimen 4, which consumed a greater proportion of elasmobranchs, in eight parameters.

**Table 1:** Results of ANOVA comparing samples from multiple different individuals with different diets (wild and captive), and samples from multiple sites within a tooth.

Parameter	F	*p*	df	B–H correction
Sq	2.7789w	0.0781	4, 11.558	
Ssk	2.0122w	0.1596	4, 11.457	
Sku	**5.5796w**	**0.0117**	4, 10.413	
Sp	1.6849	0.1825	4, 27	
Sv	1.8052w	0.1926	4, 12.021	
Sz	1.1382w	0.3873	4, 11.301	
Sds	**18.8940**	**0.0001**	4, 27	Significant
Str	**3.5685w**	**0.0393**	4, 11.806	
Sal	**8.8923**	**0.0001**	4, 27	Significant
Sdq	**7.1046w**	**0.0037**	4, 11.855	Significant
Ssc	**3.8799w**	**0.0289**	4, 12.433	
Sdr	**7.3744w**	**0.0030**	4, 12.069	Significant
Vmp	2.0855w	0.1505	4, 11.143	
Vmc	2.5595w	0.0918	4, 12.24	
Vvc	2.4292w	0.1042	4, 12.147	
Vvv	3.1651w	0.0569	4, 11.334	
Spk	1.9043w	0.1802	4, 10.958	
Sk	**2.4630w**	**0.1001**	4, 12.334	
Svk	3.2954w	0.0527	4, 10.96	
Smr1	1.7856	0.1609	4, 27	
Smr2	**4.5516w**	**0.0185**	4, 11.841	
S5z	1.2853w	0.3350	4, 10.774	
Sa	2.6142w	0.0888	4, 11.902	

W indicates Welch ANOVA; significant differences (*P* < 0.05) in bold. B–H correction reports the results of a Benjamini–Hochberg procedure (False Discovery Rate 0.05).

**Table 2:** Pairwise differences (Tukey HSD) between samples from multiple different individuals with different diets (wild, specimens 2–4) and captive (specimen 1), and samples from multiple sites within a tooth (samples 2a).

4	Differs from	1, 2, 2a	Sds
4	Differs from	1, 2a	Sq, Sal, Vmp, Vvv, Spk, Svk
4	Differs from	1	Sv, Sz, Vmc, Vvc, Sk, S5z, Sa
3	Differs from	1, 2a	Sds, Sal
2	Differs from	1	Sds

**Table 3:** Pairwise differences (Tukey HSD) between samples from multiple different individuals with different diets (wild specimens 2–4 and captive (specimen 1), and samples from multiple sites within a tooth (samples 2a).

	Sample 1	Sample 2a	Specimen 2	Specimen 3	Specimen 4
Sample 1	—		Sds	Sds, Sal	Sq, Sv, Sz, Sds, Sal, Vmp, Vmc, Vvc, Vvv, Spk, Sk, Svk, S5z, Sa
Sample 2a	0	—		Sds, Sal	Sq, Sds, Sal, Vmp, Vvv, Spk, Svk
Specimen 2	1	0	—		Sds
Specimen 3	2	2	0	—	
Specimen 4	14	7	1	0	—

The same ANOVA allows us to reject the null hypothesis that microwear does not vary with diet in *C. taurus*. Four parameters differ significantly between specimens (Table 1; after application of a Benjamini–Hochberg procedure to account for multiple comparisons). Pairwise comparisons (Tukey HSD; Tables 2 and 3) reveal differences are greatest between sample 1 (teeth from aquarium sharks fed fish only) and sample 4 (western Atlantic individual, larger than the ca. 2 m threshold for increased consumption of elasmobranchs). The number of pairwise differences between specimens increases with dietary difference (Table 3). ANOVA based only on specimens with different diets (i.e., excluding the within tooth samples, 2a) yields a very similar result (Supplementary Tables S2–S4 online; five parameters exhibit significant differences after application of a Benjamini–Hochberg procedure). Subsampling to compare six random teeth from the wild specimens with the aquarium sample found significant differences in every sub-sampling ANOVA (Supplementary Table S5 online): nine parameters were significant in more than 50% of sub-sample sets. Lower left area of table shows the number of pairwise differences; upper right shows the parameters that differ. 

**Figure 1:**
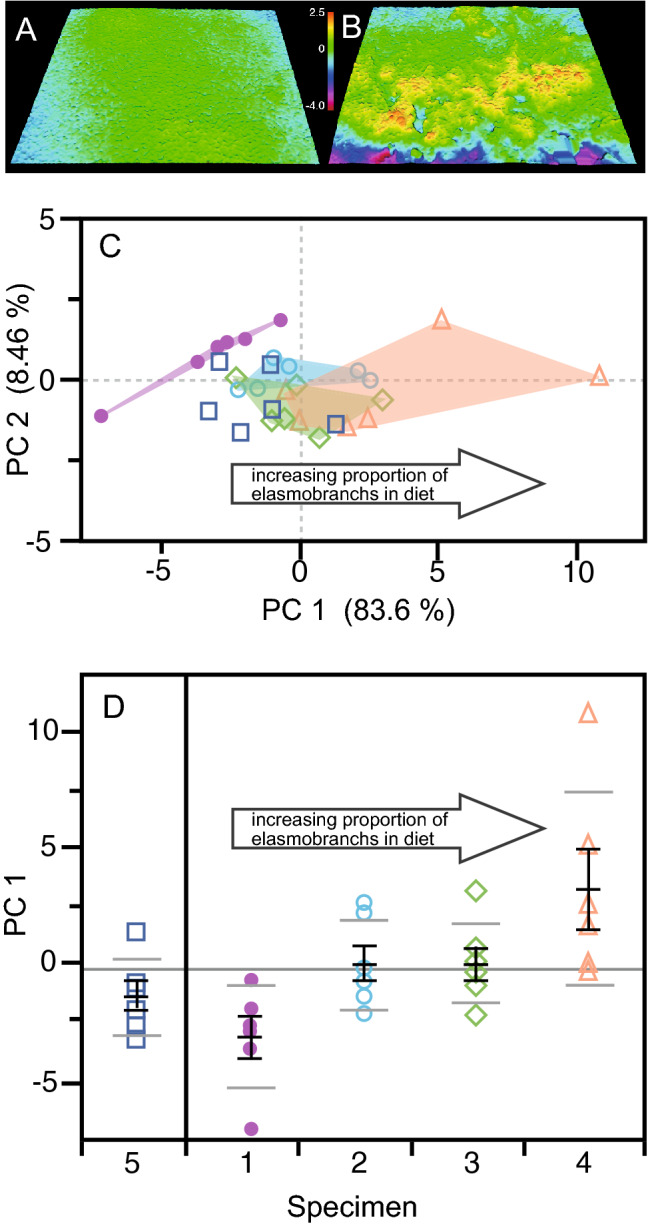
Tooth microwear textures of *Carcharias taurus*, and principal components analysis of International Organization for Standardization (ISO) texture parameters. **A**, **B** Digital elevation models showing levelled surface data (146 µm × 110 µm) for samples from an aquarium fed individual with a fish-only diet (**A** specimen 1d: LEIUG 123404), and a wild individual > 2 m in length (in wild populations individuals of this size consume higher proportions of elasmobranchs (**B** specimen 4:CT001). **C** PCA of 14 ISO parameters that exhibit significant pairwise differences between elasmobranchs with different diets. PC1 is strongly correlated with diet, with higher scores linked to wild individuals which, based on available evidence^30–32^, consumed higher proportions of elasmobranch prey in the diet, and a greater range of prey types. Figure is based on data from specimens 1–4. **D** PCA scores by specimen, showing mean and standard error (black bars) and 95% confidence intervals on means; Horizontal line across plot in grand mean. Both means and variances of PC1 values are correlated with diet (Rs = 1). Specimen 5, with unknown diet, displays PC1 scores most similar to those of individuals with a teleost dominated diet.

Fourteen parameters exhibit pairwise differences between samples (Table 3), and PCA of the elasmobranchs with different diets (i.e. excluding the within tooth samples, 2a) based in these parameters reveals a clear pattern (Fig. 1). PC 1 captures 83.6% of the variance and is significantly correlated with diet (*R*_*s*_ = 0.41252; *P* = 0.0007); the mean and range of PC 1 values increases as the proportion of elasmobranchs and the diversity of prey increases (increasing from sample 1—aquarium-fed fish-only diet—to sample 4—highest proportion of elasmobranchs). Translating the technical characteristics of texture into more comprehensible language is not straightforward, but as the proportion of elasmobranchs and diversity of prey consumed increases, tooth surface textures become what might be considered in general terms to be ‘rougher’ (see Ref.^24^ for discussion of the relationship between qualitative evaluations of roughness and texture parameters), with samples displaying greater variation in texture between the teeth. All but one of the parameters that differ increase: the height of surface texture increases (Sq, Sa, Sz), as does the height of the core of the surface (Sk), and the volume of material and voids in the core and valleys (Vmc, Vvc, Vvv). The height and volume of material in peaks increase with consumption of elasmobranchs (S5z, Spk, Vmp), but Sds decreases, indicating that peaks make up a smaller proportion of the area of surfaces.

PC 2 is also correlated with diet, but to a lesser degree (*R*_*s*_ =  − 0.257156; *P* = 0.0114). Neither PC 1 nor PC 2 is correlated with body length (PC 1: *R*_*s*_ = 0.05144, *P* = 0.2866; PC 2: *R*_*s*_ = 0.00004, *P* = 0.9758).

Projecting sample 5 into the PCA (Fig. 1) reveals that its microwear textures are comparable to those of samples 1 and 2. ANOVA of PC 1 scores reveals significant differences (F = 5.2296, d.f. 4, 25, *P* = 0.0034) between samples, and pairwise testing (Tukey HSD) indicates that sample 5 does not differ from samples 1, 2 and 3, and that samples 1, and 5 both differ from sample 4.

## Discussion

Given that elasmobranchs are well known for the rate at which they replace their teeth, it is perhaps surprising that anterior teeth are retained long enough for dietarily informative microwear textures to develop. Yet our results demonstrate that tooth microwear textures vary with diet in *C. taurus*, and show that DMTA can provide an additional, potentially powerful tool for dietary discrimination in elasmobranchs. Furthermore, recent analysis indicates that *C. taurus* mostly consume prey in one piece^30^, implying less interaction of teeth with prey than would the case in animals that process their food before swallowing. We predict that for elasmobranchs that bite their prey the relationship between diet and microwear texture will be even stronger than that reported here.

Sampling individuals with different diets reveals increases in PC 1 values that in turn correspond to changes in a number of different ISO texture parameters. In general terms, as noted above, there is a trend towards ‘rougher’ surfaces with increases in the proportion of elasmobranchs in *C. taurus *diets, and with increasing consumption of benthic elasmobranchs^30–32^ (which may be associated with an increase in the amount of sediment consumed with prey). The increase in variance of PC1 values may also reflect increased diversity of prey types^30–32^ in larger individuals. To a degree, the greater variance might reflect the greater difference between maximum development of ‘rough’ microwear texture in a tooth near the end of its functional life compared to a smooth, recently erupted tooth. Either way, our results indicate that microwear texture tracks diet, but more work will be required to tease apart these additional factors.

Our analyses indicate that the tooth microwear textures of Specimen 5, from a different geographic area to other specimens, and for which we have no dietary data, are closely comparable to those of samples 1, 2 and 3, in terms of both values and variances. On this basis we interpret specimen 5 to have had a diet dominated by fish. The larger size of this specimen (at ca. 335 cm, larger than any other specimens analysed) lends further support to the hypothesis that microwear texture is tracking diet, and not size. Our dietary predictions regarding *C. taurus* from this area could be tested using traditional stomach contents, or stable isotope analyses, but this is outside the scope of the present study.

Our results also suggest that application of DMTA to analysis of the diet of individual sharks will produce more reliable results if multiple teeth are sampled rather than a single tooth. Comparing the six teeth of the aquarium individuals (fed only fish) with six teeth sampled randomly from the wild individuals (which had more varied diets) revealed significant differences in every sub-sampling (Supplementary Table S5). However the number of parameters displaying a significant difference between wild and aquarium teeth varied, and fewer significant differences than were found than analyses comparing the aquarium teeth to multiple teeth from each wild individual. This suggests that analyses based on single isolated teeth rather than those from jaws, a situation that would commonly arise in analyses of fossil teeth, have the potential to detect differences between populations and species with different diets, but will be less sensitive than analyses based on multiple teeth per individual. To a certain extent, this will be offset in collections of isolated fossil teeth because the vast majority are teeth that were shed at the end of the functional cycle, so there will be much less sampling of recently erupted teeth with less well-developed microwear textures. (Due to the rate of tooth replacement in elasmobranchs, the number of teeth shed by an individual in its lifetime outnumber the number of teeth in the individuals jaw at time of death by several orders of magnitude).

Drawing wider comparisons with microwear texture analyses in other groups of vertebrates, of the relationship between diet and 3D microwear texture based on ISO parameters, the number of parameters that differ between samples of *C. taurus* is larger than most previous studies, probably due to greater differences in material properties of food between the samples compared. Wild *C. taurus* consume a wider variety of prey than aquarium fed *C. taurus.* Wild individuals consume ‘harder’ prey items, whilst interacting with the natural environment. A wild individual consuming a benthic elasmobranch will have to bite through dermal denticles, a larger cartilage skeleton and inevitably will ingest some sediment during the process. In contrast aquarium individuals are largely fed whole and partial fish within the water column, a much ‘softer’ diet. Comparison of this study to others analysing vertebrate diet, repeatedly display significant differences in certain parameters when comparing groups with harder/softer diets. Purnell and Darras^23^ found that Sdq, Sdr, Vmc, Vvv, Sk and Sa discriminated best between the specialist durophagous and more opportunist durophagous fish in their study (based on ANOVA and PCA), with these parameters also differing between populations of the opportunist durophage *Archosargus probatocephalus* with different proportions of hard prey in their diets. Of these parameters, Sk, Sa, Vmc, and Vvv produce pairwise differences between *C. taurus* samples (between 1 and 4). These parameters capture aspects of surface heights and the volumes of material within the core and voids in valleys, respectively (Supplementary Table S1 online). All increase in value as the proportion of elasmobranchs in the diet increases, the same as the pattern of increase with durophagy seen in *Archosargus probatocephalus* and *Anarhichas lupus*^23^. Vmc, Vvv, and Sk were also found to increase with the amount of hard-shelled prey in the diet of cichlids^24^. This means that ‘harder’ diets produce tooth surface textures with greater core depth and an increase in the volumes of core material and valleys. In short ‘harder’ diets produce rougher tooth surfaces.

This conclusion is also supported by a recent DMTA study on reptiles^29^, which exhibit significant overlap with sharks in the parameter trends correlating with ‘harder’ diets. Of the parameters correlating with increasing PC 1 values in sharks, parameters correlated with increasing dietary ‘hardness’ in reptiles include those capturing aspects of texture height (Sa, Sq, S5z), the number of peaks (Spk), and the depth, void volume and material volume of the core (Sk, Vvc, Vmc). Once again ‘harder’ diets produce rougher tooth surfaces.

Other studies, although focussed on terrestrial rather than aquatic vertebrates, have found similar patterns. Vmc, Vvc, Vvv, and Sa increase with more abrasive diets in grazing ungulate mammals^34^; Vmc, Vvv and Sk increase with increasingly ‘hard’ prey in insectivorous bats^21^. Unlike other studies, the latter found Sa (the average surface height) to decrease with harder diets^26^. A recent study of bats and moles^35^ found that, like sharks, increasing the ‘hardness’ of the prey creates rougher tooth surfaces that can be defined by increases in Sa, Vmc, VVc values (amongst others) and a decrease in Sds values (amongst others).

## Conclusions

Our study of *C. taurus* and comparisons with previous work demonstrate that dental microwear texture analysis (DMTA) can provide an additional, potentially powerful tool for dietary discrimination in elasmobranchs. Even though our sample size is small, DMTA provides significant results; it is applicable to individuals with no gut contents, and thus offers a potential means to reduce the impact on wild elasmobranch populations when analysing their dietary ecology (especially relevant in conservation of endangered elasmobranch species). Because microwear accumulates over longer periods of time DMTA also overcomes the ‘snapshot bias’ of stomach contents analysis. In combination with other approaches that capture dietary information on different timescales^33^, it could be used as part of a multiproxy approach to dietary analysis in elasmobranchs. In an evolutionary context, DMTA applied to extinct elasmobranchs has the potential to provide a robust new approach to testing hypotheses of dietary ecology, niche segregation and escalation through a fossil record of teeth and jaws spanning 400 million years.

## Materials and methods

### Materials and sampling strategy

To provide baseline data from individuals known to consume only fishes, six shed anterior teeth of captive *C. taurus* were obtained by divers from tanks at Sea Life London Aquarium (specimens 1a–1f.; Table 4). These teeth are derived from four individuals that were approximately 270 cm in length, and were fed on a controlled diet of whole ‘white fish’ and occasional tropical *Caranx *sp. The specific individual from which each tooth originated is unknown. Analysis of tooth loss rate indicates that a single tooth is in use for 80–90 days before being shed^36^; all aquarium teeth sampled were shed naturally at the end of a replacement cycle.

**Table 4:** Specimens of *Carcharias taurus* from which tooth texture data were obtained.

Repository number	Specimen number (this analysis)	Locality	Date of capture	Specimen size	Diet (relative proportion of elasmobranchs)
LEIUG 123402	Specimen 1a	Sea Life London Aquarium		~ 270 cm	Fish only
LEIUG 123403	Specimen 1b				
LEIUG 123404	Specimen 1c				
LEIUG 123405	Specimen 1d				
LEIUG 123406	Specimen 1e				
LEIUG 123407	Specimen 1f				
Florida Museum of Natural History UF47900	Specimen 2	Tanzler Waters Reef, Florida	03/1981	~ 190 cm	Fish, relatively small proportion of elasmobranch
Florida Museum of Natural History 19,705,007.17	Specimen 3	Florida 29°06.77 N 80°49.73 W	01/081997	240 cm measured at capture	Fish, intermediate proportion of elasmobranch
Florida Museum of Natural History	Specimen 4	North Carolina 35°09 N 75°47 W	17/04/1975	278 cm measured at capture	Fish, relatively larger proportion of elasmobranch
Private collection (Gordon Hubbell) GH-CT-P-12	Specimen 5	Cebu City, Philippines		~ 335 cm size estimated at capture	Unknown

Dietary preferences for specimens 2–4 were inferred from published analyses^30–32^demonstrating that in wild populations of the Western Atlantic^31,32^ larger *C. taurus* consume more elasmobranchs than smaller individuals (< 2 m total length).

Teeth from wild individuals were sampled in jaws of *C. taurus* captured in the western Atlantic, off the east coast of the USA (Specimens 2–4; Table 4). Individuals ranged in length from 190 to 278 cm. Previous studies^30–32^, including populations from the Western Atlantic, indicate that diet differs with size: larger individuals (> 2 m) consume a greater proportion of elasmobranchs (particularly benthic species) and a greater diversity of prey. Our sample includes both smaller (< 2 m) and larger (> 2 m) *C. taurus*.

An additional specimen captured in the Western Pacific and landed in the Philippines was also analysed (specimen 5). No dietary data are available for this specimen, or this population, but microwear data were used to test the hypothesis that textures in wild sharks vary purely as consequence of size, rather than diet.

The data used to test the hypothesis that non-dietary variation in microwear texture between samples from different parts of a tooth is greater than variation between individuals with different diets were obtained from an anterior tooth from specimen 2. Eight samples were obtained from positions ranging across the labial surface of the tooth. For all other tests, data were collected from the central part of the labial surface, approximately 5 mm below the apex of the tooth.

The data used to test the hypothesis that microwear texture varies with diet were sampled from the six aquarium teeth (known, from their morphology, to be from anterior locations in the jaw), and six teeth per individual from the wild western Atlantic specimens (individuals 2–4). These teeth were selected at random from among the six anterior most teeth of the outer tooth row of the upper jaw and the equivalent teeth on the lower jaw. The same sampling strategy was applied to Specimen 5.

### Surface texture data acquisition

Data were acquired from high fidelity surface replicas of teeth prepared using President Jet medium body polysiloxane dental moulding compound, and EpoTek 320 LV black epoxy. Both were mixed and applied following the manufacturer's instructions. Analysis of accuracy and precision of moulding compounds indicates that replicas made this way compare favourably with the most accurate and precise moulding compounds, with very small absolute differences in parameter values between replica and original^37^.

High-resolution 3D surface data were captured following standard lab protocols^23,24,37^, using an Alicona Infinite Focus microscope G4b (IFM; Alicona GmbH, Graz, Austria; software version 2.1.2), equipped with a × 100 objective to give a field of view of 146 × 111 µm. The Alicona Infinite Focus microscope G4b has a CCD of 1624 × 1232 pixels. In theory, for a field of view of 146 µm, this equates to a lateral sampling distance of 0.09 µm, but the limits imposed by the wavelength of white light mean that lateral optical resolution is between 0.35 and 0.4 µm. For all samples, vertical and lateral resolutions were set at 20 nm and 440 nm respectively. Exposure settings were manually adjusted to maximize data quality (between 7.18 and 6.5 ms); contrast was set at 2.0. Point clouds were edited manually to delete measurement errors (e.g., single point data spikes) and extraneous dirt and dust particles from the surface. After editing, point clouds were exported as .sur files and imported into SurfStand (software version 5.0; restore bad data option selected). Surfaces were then treated by levelling the surface and removing gross tooth form with a second order polynomial function, and applying a spline filter, with a nesting index of 0.025 mm. The resulting scale limited roughness surface was then used for calculation of ISO 25178-2 standard parameters^38^, quantifying tooth surface texture (Supplementary Table S1 online).

### Statistical analysis

The texture data are normally distributed (Shapiro-Wilks tests) so parametric tests were used except for analysis of relationships between texture and non-normal data (dietary rank and length data for elasmobranchs). Hypotheses were explored using analysis of variance (ANOVA), correlations (Spearman’s Rank), pairwise testing (Tukey HSD), and principal components analysis (on correlations; PCA). Where homogeneity of variance tests (Bartlett and Levene tests) revealed evidence of unequal variances, Welch ANOVA was used. A Benjamini–Hochberg procedure was used to account for the possibility of inflated Type I error rates associated with multiple comparisons. The False Discovery rate was set at 0.05.

Two sets of analyses were performed to test the hypothesis that texture varies with diet. The first was a simple ANOVA and pairwise tests of the sample of teeth from aquarium elasmobranchs fed a fish-only diet, and the wild, western Atlantic specimens (3 samples, 6 teeth in each). Because the nature of sampling in the aquarium and wild datasets differs (in the aquarium, teeth represent random sampling of anterior teeth from multiple individuals, whereas each of the wild samples is random but from within an individual) a second set of tests was conducted to simply compare the six aquarium teeth with subsets of six randomly sampled teeth from the wild, western Atlantic specimens. This simulated, for the wild specimens, the random sampling of shed teeth from the aquarium. This sampling process was repeated 10 times (Supplementary Table S5 online). PCA was based on the parameters found to differ in the ANOVA and Tukey HSD tests conducted on the aquarium and three western Atlantic sharks. The diet of specimen five was evaluated by projecting samples into this PCA. All statistical tests were carried out using JMP, versions 12 (SAS Institute, Cary, NC, USA).

### Sample acquisition

All wild caught specimens investigated within this study were in the form of dried jaws housed in museum collections. No capture of wild specimens was undertaken as part of this study.

## Supplementary information

Supplementary Tables.

## Data Availability

The texture parameters, derived from scale limited surfaces, upon which this study is based are available at: 10.25392/leicester.data.13299161.
