# Supplementary material for: Dental microwear texture analysis as a tool for dietary discrimination in elasmobranchs
Source: Sci Rep. 2021 Jan 28;11:2444. doi: 10.1038/s41598-021-81258-9 (PMC7844039; doi:10.1038/s41598-021-81258-9)
Supplement: Supplementary file 1 — Supplementary Tables. [file 41598_2021_81258_MOESM1_ESM.pdf]

Supplementary Information for:

Dental microwear texture analysis as a tool for dietary discrimination in elasmobranchs

Laura J. McLennan[†*], and Mark A. Purnell[*]

University of Leicester, Centre for Palaeobiology Research, School of Geography, Geology and the Environment, , Leicester, LE1 7RH, UK; [†]Current address: Department of Environmental Science, University of Derby, Derby, DE22 1GB, UK

* Correspondence to: Laura J Mclennan <l.mclennan@derby.ac.uk>, Mark A Purnell <map2@leicester.ac.uk>

Supplementary Information

Supplementary table S1: Short definitions and categorization of 3D areal surface texture parameters. For further explanation see Bestwick et al. (2019) and ISO 25178-2 (International Orgnisation for Standardization 2012).

parameter	unit	definition	
Sq	µm	Root-Mean-Square height of surface	height
Sp	µm	Maximum peak height of surface	height
Sv	µm	Maximum valley depth of surface	height
Sz	µm	Maximum height of surface	height
Sa	µm	Average height of surface	height
Ssk	-	Skewness of height distribution of surface	height
Sku	-	Kurtosis of height distribution of surface	height
S5z	µm	10 point height of surface	feature
Sdq	-	Root mean square gradient of the surface	hybrid
Sdr	%	Developed interfacial area ratio	hybrid
Sds	$1/mm^2$	Density of summits. Number of summits per unit area making up the surface	hybrid
Ssc	1/µm	Mean summit curvature for peak structures	
Sk	µm	Core roughness depth, Height of the core material	material ratio
Spk	µm	Mean height of the peaks above the core material	material ratio
Svk	µm	Mean depth of the valleys below the core material	material ratio
Smr1	%	Surface bearing area ratio (the proportion of the surface which consists of peaks above the core material)	material ratio
Smr2	%	Surface bearing area ratio (the proportion of the surface which would carry the load)	material ratio
Vmp	$µm^3/mm^2$	Material volume of the peaks of the surface	volume
Vmc	$µm^3/mm^2$	Material volume of the core of the surface	volume
Vvc	$µm^3/mm^2$	Void volume of the core of the surface	volume
Vvv	$µm^3/mm^2$	Void volume of the valleys of the surface	volume
Sal	mm	Auto correlation length. Horizontal distance of the auto correlation function (ACF) which has the fastest decay to the value 0.2. Large value: surface dominated by low frequencies. Small value: surface dominated by high frequencies.	spatial
Str	-	Texture aspect ratio (values range 0-1). Ratio from the distance with the fastest to the distance with the slowest decay of the ACF to the value. 0.2-0.3: surface has a strong directional structure. > 0.5: surface has rather uniform texture.	spatial

Supplementary table S2: Results of ANOVA comparing samples from multiple individuals with different diets (wild and captive; specimens 1 - 4), ^{w} indicates Welch ANOVA; significant differences (P<0.05) in bold. B-H correction reports the results of a Benjamini-Hochberg procedure (False Discovery Rate 0.05).

Parameter	F	*p*	df	B-H correction
**Sq**	**5.6677**	**0.0056**	3, 20	Significant
**Ssk**	1.1095	0.3686	3, 20	
**Sku**	1.0342	0.3988	3, 20	
**Sp**	1.7329	0.1925	3, 20	
**Sv**	2.1293^{w}	0.1622	3, 9.6444	
**Sz**	2.3187	0.1063	3, 20	
**Sds**	**21.5572**	**0.0001**	3, 20	Significant
**Str**	1.6460	0.2106	3, 20	
**Sal**	**6.8788**	**0.0023**	3, 20	Significant
**Sdq**	1.5501^{w}	0.2604	3, 10.279	
**Ssc**	**3.8640^{w}**	**0.0445**	3, 10.144	
**Sdr**	1.3992^{w}	0.2977	3, 10.374	
**Vmp**	**3.5550**	**0.0328**	3, 20	
**Vmc**	3.5150^{w}	0.0556	3, 10.296	
**Vvc**	3.3413^{w}	0.0624	3, 10.356	
**Vvv**	**5.3667**	**0.0071**	3, 20	Significant
**Spk**	**3.5394**	**0.0333**	3, 20	
**Sk**	3.3593^{w}	0.0641	3, 9.8549	
**Svk**	**4.5676**	**0.0136**	3, 20	
**Smr1**	1.1868	0.3400	3, 20	
**Smr2**	1.9279	0.1576	3, 20	
**S5z**	2.6399	0.0775	3, 20	
**Sa**	**5.7277**	**0.0054**	3, 20	Significant

Supplementary table S3: Pairwise differences (Tukey HSD) between samples from multiple individuals with different diets (wild; specimens 2 - 4) and aquarium sharks (sample 1; fish-only diet).

1	differs from	2,3,4	Sds
1	differs from	3, 4	Sal
1	differs from	4	Sq, Vmp, Vmc, Vvc, Vvv, Spk, Sk, Svk, Sa
2	differs from	4	Sds

Supplementary table S4: Pairwise differences (Tukey HSD) between samples from multiple individuals with different diets (wild; specimens 2 - 4) and aquarium sharks (sample 1; fish-only diet). Lower left side of matrix tallies differences, upper right showing the parameters that differ.

	**Sample 1**	**Specimen 2**	**Specimen 3**	**Specimen 4**
**Sample 1**		Sds	Sds, Sal	Sq, Vmp, Vmc, Vvc, Vvv, Spk, Sk, Svk, Sa, Sds, Sal
**Specimen 2**	1			Sds
**Specimen 3**	2	0		
**Specimen 4**	11	1	0	

Supplementary table S5: Results of ANOVA testing the hypothesis that microwear textures on six randomly subsampled wild teeth do not differ from those of the six teeth from aquarium specimens of *C. taurus* (^{w} indicates Welch ANOVA; significant differences (p<0.05) in bold).

Parameter	***Sub-sample 1***			***Sub-sample 2***			***Sub-sample 3***			***Sub-sample 4***			***Sub-sample 5***		
	**F**	***p***	**df**	**F**	***p***	**df**	**F**	***p***	**df**	**F**	***p***	**df**	**F**	***p***	**df**
**Sq**	**7.5273**	**0.0207**	1, 10	**8.2959**	**0.0164**	1, 10	**5.4979**	**0.0410**	1, 10	**5.0335**	**0.0487**	1, 10	6.1554w	0.0526	1, 5.349
**Ssk**	1.6821	0.2238	1, 10	0.9779	0.3460	1, 10	2.8132	0.1244	1, 10	1.0941	0.3202	1, 10	3.9191	0.0759	1, 10
**Sku**	0.6401	0.4423	1, 10	0.0000	0.9986	1, 10	0.3212^{w}	0.5886	1, 6.996	0.1141	0.7425	1, 10	0.1230	0.7331	1, 10
**Sp**	2.6954	0.1317	1, 10	0.2650	0.6179	1, 10	1.4522	0.2559	1, 10	0.2569	0.6232	1, 10	0.1089w	0.7522	1, 6.301
**Sv**	**7.5794**	**0.0204**	1, 10	2.8608	0.1216	1, 10	**5.2689**	**0.0446**	1, 10	1.1881	0.3013	1, 10	**5.0533**	**0.0483**	1, 10
**Sz**	**5.2584**	**0.0448**	1, 10	1.4440	0.2572	1, 10	3.4150	0.0944	1, 10	0.7161	0.4172	1, 10	2.1109	0.1769	1, 10
**Sds**	**36.8229**	**0.0001**	1, 10	**31.0817^{w}**	**0.0014**	1, 6.075	**19.2529^{w}**	**0.0053**	1, 5.677	**67.6199**	**0.0001**	1, 10	**31.9545w**	**0.0012**	1, 6.192
**Str**	3.4110	0.0945	1, 10	4.8400	0.0524	1, 10	1.2809^{w}	0.3011	1, 5.978	3.8536	0.0780	1, 10	2.4649w	0.1651	1, 6.303
**Sal**	**16.2000**	**0.0024**	1, 10	**8.1818**	**0.0169**	1, 10	**16.2000**	**0.0024**	1, 10	**18.8462**	**0.0015**	1, 10	**9.8000**	**0.0107**	1, 10
**Sdq**	1.0289	0.3343	1, 10	0.5989	0.4569	1, 10	0.4786	0.5048	1, 10	0.0095^{w}	0.9260	1, 5.411	0.0027	0.9597	1, 10
**Ssc**	0.1146	0.7419	1, 10	0.2407	0.6343	1, 10	0.0125	0.9132	1, 10	1.7855	0.2111	1, 10	1.8029	0.2090	1, 10
**Sdr**	0.9052	0.3638	1, 10	0.5476	0.4763	1, 10	0.3122	0.5887	1, 10	0.1324^{w}	0.7295	1, 5.515	0.0153	0.9041	1, 10
**Vmp**	4.9505	0.0503	1, 10	4.6831	0.0557	1, 10	2.8036	0.1250	1, 10	2.0230	0.1854	1, 10	1.7351	0.2171	1, 10
**Vmc**	**8.1668**	**0.0170**	1, 10	**8.9663**	**0.0135**	1, 10	**7.0975**	**0.0237**	1, 10	**5.0163**	**0.0490**	1, 10	5.4327w	0.0607	1, 5.714
**Vvc**	**8.1852**	**0.0169**	1, 10	**9.0758**	**0.0131**	1, 10	**6.4194**	**0.0297**	1, 10	4.7692	0.0539	1, 10	5.3855w	0.0619	1, 5.662
**Vvv**	**5.9782**	**0.0345**	1, 10	**5.6400**	**0.0390**	1, 10	3.8972^{w}	0.0931	1, 6.358	4.1442	0.0691	1, 10	**6.1870**	**0.0321**	1, 10
**Spk**	4.7055	0.0552	1, 10	3.8691	0.0775	1, 10	2.6770	0.1329	1, 10	1.7714	0.2128	1, 10	1.4473	0.2567	1, 10
**Sk**	**8.3521**	**0.0161**	1, 10	**9.5260**	**0.0115**	1, 10	**7.8068**	**0.0190**	1, 10	5.0210^{w}	0.0646	1, 6.236	5.3829w	0.0595	1, 5.997
**Svk**	**5.6671**	**0.0386**	1, 10	4.5509	0.0587	1, 10	3.7126^{w}	0.1015	1, 6.097	3.2091	0.1035	1, 10	**6.0905**	**0.0332**	1, 10
**Smr1**	0.1399^{w}	0.7189	1, 7.41	0.5570	0.4726	1, 10	0.1337	0.7222	1, 10	0.0336	0.8583	1, 10	0.0531	0.8224	1, 10
**Smr2**	0.0139	0.9086	1, 10	0.8460	0.3793	1, 10	0.0715	0.7946	1, 10	0.2684	0.6157	1, 10	0.3491	0.5677	1, 10
**S5z**	4.5638	0.0584	1, 10	1.7583	0.2143	1, 10	2.3585	0.1556	1, 10	0.7926	0.3942	1, 10	1.2556w	0.3058	1, 5.938
**Sa**	**7.9504**	**0.0182**	1, 10	**8.9876**	**0.0134**	1, 10	**6.2075**	**0.0319**	1, 10	**5.0876**	**0.0477**	1, 10	5.8589w	0.0569	1, 5.343

Parameter	*Sub-Sample 6*			*Sub-sample 7*			*Sub-sample 8*			*Sub-sample 9*			*Sub-sample 10*		
	**F**	***p***	**df**	**F**	***p***	**df**	**F**	***p***	**df**	**F**	***p***	**df**	**F**	***p***	**df**
**Sq**	4.1013	0.0704	1, 10	**9.7333**	**0.0109**	1, 10	**5.5465**	**0.0403**	1, 10	**5.7745**	**0.0371**	1, 10	**6.5220**	**0.0287**	1, 10
**Ssk**	1.2554	0.2887	1, 10	2.4744	0.1468	1, 10	1.3809	0.2672	1, 10	0.9556	0.3514	1, 10	3.5344	0.0895	1, 10
**Sku**	0.0413^{w}	0.8445	1, 7.393	0.6815	0.4283	1, 10	**1.2046^{w}**	**0.3084**	1, 7.068	1.1937	0.3002	1, 10	0.9347^{w}	0.3665	1, 6.864
**Sp**	0.5376	0.4803	1, 10	1.3588	0.2708	1, 10	0.9275	0.3582	1, 10	1.9887	0.1888	1, 10	1.4238	0.2603	1, 10
**Sv**	2.8624	0.1215	1, 10	**6.8390**	**0.0258**	1, 10	2.9620	0.1160	1, 10	2.7157	0.1304	1, 10	**4.9911**	**0.0495**	1, 10
**Sz**	1.6058	0.2338	1, 10	4.0081	0.0731	1, 10	1.9038	0.1977	1, 10	2.4713	0.1470	1, 10	3.1631	0.1057	1, 10
**Sds**	**31.476^{w}**	**0.0013**	1, 6.075	**41.4737**	**0.0001**	1, 10	**43.255^{w}**	**0.0005**	1, 6.230	**32.773^{w}**	**0.0013**	1, 5.972	**48.3854**	**0.0001**	1, 10
**Str**	1.8284^{w}	0.2260	1, 5.878	**11.0907**	**0.0076**	1, 10	3.1500^{w}	0.1197	1, 6.913	3.1488	0.1064	1, 10	5.5360^{w}	0.0567	1, 6.026
**Sal**	**6.4811^{w}**	**0.0250**	1, 6.481	**8.1818**	**0.0169**	1, 10	**10.9459**	**0.0079**	1, 10	**16.2000**	**0.0024**	1, 10	**35.5882**	**0.0001**	1, 10
**Sdq**	0.3674	0.5579	1, 10	0.5585	0.4721	1, 10	0.2403	0.6346	1, 10	0.1601	0.6975	1, 10	0.1776	0.6823	1, 10
**Ssc**	0.1431	0.7131	1, 10	0.2297	0.6421	1, 10	0.5790	0.4643	1, 10	0.2064	0.6593	1, 10	0.3130	0.5882	1, 10
**Sdr**	0.2201	0.6490	1, 10	0.5243	0.4856	1, 10	0.1165	0.7399	1, 10	0.0420	0.8417	1, 10	0.0663	0.8020	1, 10
**Vmp**	2.0729	0.1805	1, 10	**5.2845**	**0.0443**	1, 10	3.5260^{w}	0.1066	1, 6.374	4.2768	0.0655	1, 10	3.2598^{w}	0.1184	1, 6.344
**Vmc**	**5.2433**	**0.0450**	1, 10	**8.8337**	**0.0140**	1, 10	**6.0967**	**0.0332**	1, 10	**5.9664**	**0.0347**	1, 10	**7.2790**	**0.0224**	1, 10
**Vvc**	**5.0918**	**0.0477**	1, 10	**8.6754**	**0.0146**	1, 10	**6.2178**	**0.0318**	1, 10	**6.3395**	**0.0305**	1, 10	**6.9224**	**0.0251**	1, 10
**Vvv**	2.8831^{w}	0.1379	1, 6.319	**10.8943**	**0.0080**	1, 10	4.1697^{w}	0.0847	1, 6.339	4.6215	0.0571	1, 10	**5.6270**	**0.0391**	1, 10
**Spk**	1.8879	0.1994	1, 10	4.7723	0.0538	1, 10	3.2071	0.1036	1, 10	4.1131	0.0700	1, 10	3.0526	0.112	1, 10
**Sk**	**6.1203**	**0.0329**	1, 10	**8.1077**	**0.0173**	1, 10	**6.6785**	**0.0272**	1, 10	**6.0844**	**0.0333**	1, 10	**8.0590**	**0.0176**	1, 10
**Svk**	2.5719^{w}	0.1590	1, 6.110	**9.8839**	**0.0104**	1, 10	3.9119^{w}	0.0947	1, 6.082	4.1845^{w}	0.0857	1, 6.134	5.4832^{w}	0.0553	1, 6.359
**Smr1**	0.1569	0.7003	1, 10	0.0604	0.8108	1, 10	0.3804	0.5512	1, 10	1.2794	0.2844	1, 10	0.0056	0.9418	1, 10
**Smr2**	0.3869	0.5479	1, 10	0.0234	0.8815	1, 10	0.0234	0.8815	1, 10	0.0281	0.8702	1, 10	0.0025	0.9610	1, 10
**S5z**	1.3639	0.2699	1, 10	3.6307	0.0858	1, 10	2.3907	0.1531	1, 10	2.6206	0.1366	1, 10	2.7503	0.1282	1, 10
**Sa**	4.7044	0.0553	1, 10	**9.4091^{w}**	**0.0120**	1, 9.902	**5.8723**	**0.0359**	1, 10	**6.0129**	**0.0341**	1, 10	**6.8897**	**0.0254**	1, 10

Supplementary table S6: Loadings of parameters onto PC axes 1 and 2 (Figure 1).

Parameter	PC 1	PC 2
Sq	0.29096	0.01654
Sv	0.26755	0.20103
Sz	0.27388	0.19996
Sds	-0.18000	0.65064
Sal	0.18642	-0.64283
Vmp	0.27865	0.05518
Vmc	0.27863	0.08711
Vvc	0.28216	0.06606
Vvv	0.28250	-0.07348
Spk	0.27945	0.06609
Sk	0.26944	0.10026
Svk	0.27849	-0.09528
S5z	0.27599	0.19059
Sa	0.28721	0.05050
